# Supplementary figures and images for: Physician and patient concordance in reporting of appropriateness and prioritization for cataract surgery
Source: PLoS One. 2021 Jun 25;16(6):e0253210. doi: 10.1371/journal.pone.0253210 (PMC8232411; doi:10.1371/journal.pone.0253210)

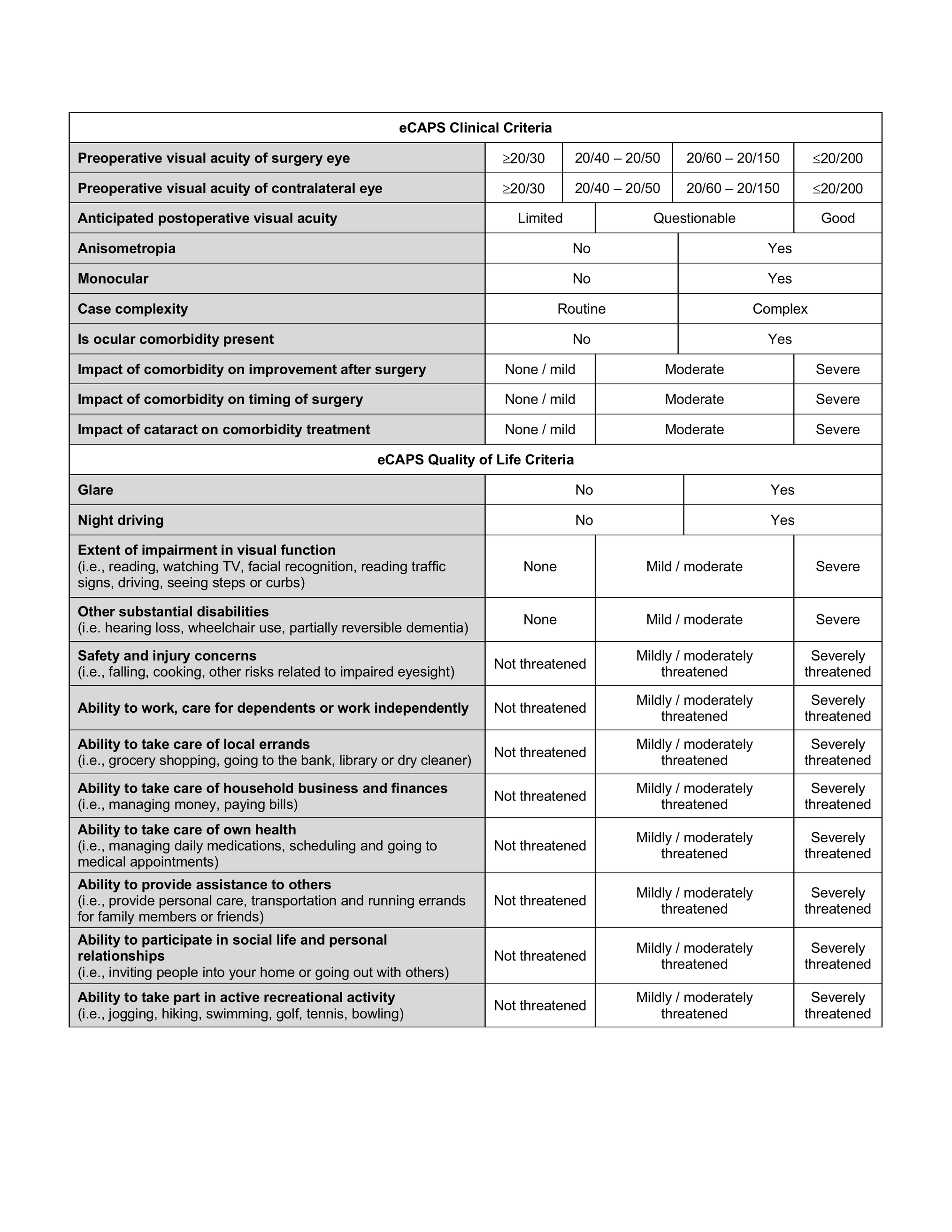

Supplement: S1 Fig — eCAPS provides a set of 10 clinical criteria scored by the surgeon and 12 quality of life measures rated on 3 levels by the patient. (TIFF) [file pone.0253210.s001.tiff]

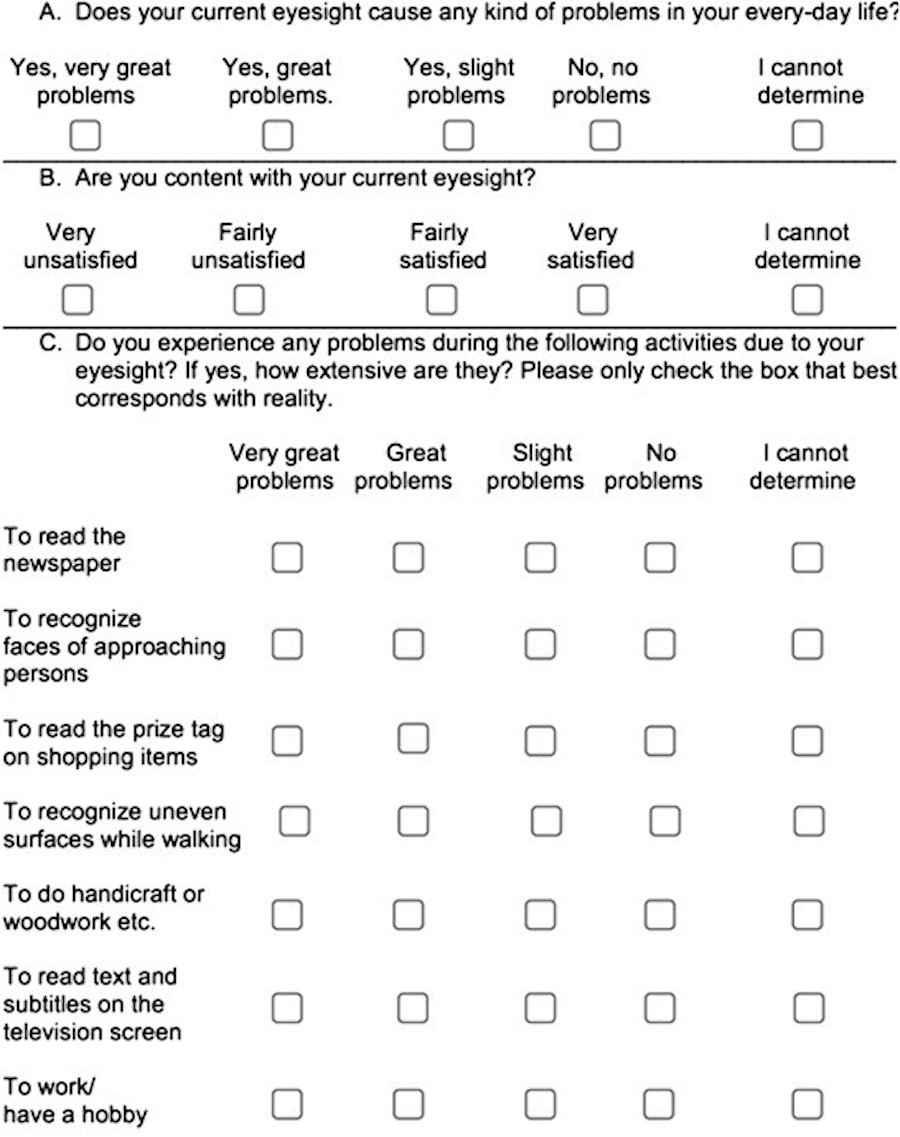

Supplement: S2 Fig — The Catquest-9SF is a Rasch-scaled, visual functioning questionnaire, consisting of two global items and seven difficulty items on a 5-point scale. (TIFF) [file pone.0253210.s002.tiff]

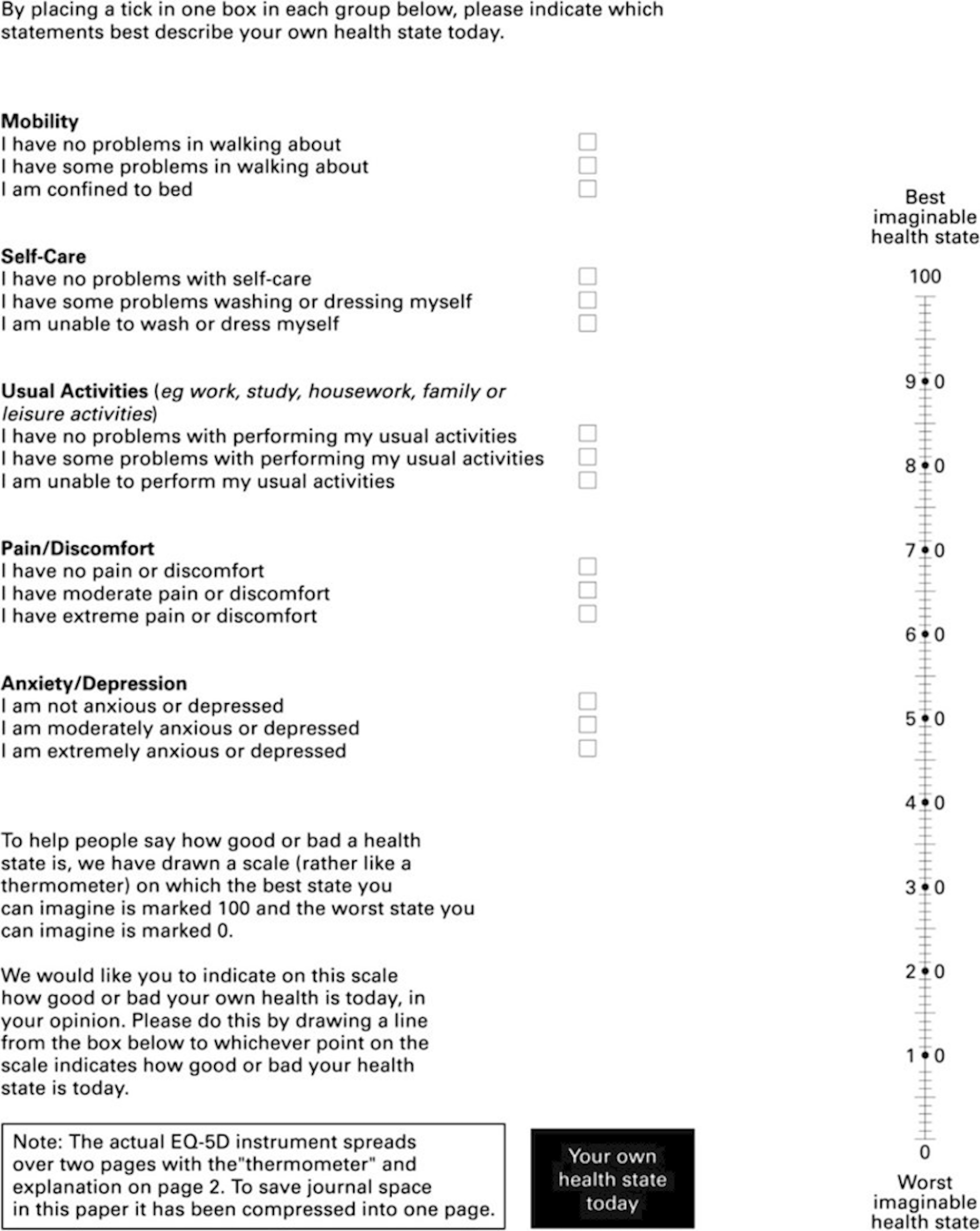

Supplement: S3 Fig — The EQ-5D is a generic health-related quality of life questionnaire, which assesses five dimensions of health including mobility, self-care, usual activities, pain/discomfort and anxiety/depression on a 5-point scale, and provides a visual analogue scale (VAS) from 0 to 100 to rank perceived overall health state. (TIFF) [file pone.0253210.s003.tiff]
