# Supplementary material for: Physician and patient concordance in reporting of appropriateness and prioritization for cataract surgery
Source: PLoS One. 2021 Jun 25;16(6):e0253210. doi: 10.1371/journal.pone.0253210 (PMC8232411; doi:10.1371/journal.pone.0253210)
Supplement: S1 Table — *Physician rating has been accounted for in each regression analysis for appropriateness. Statistically significant parameters are highlighted in yellow (by overall model F-test and parameter specific t-test). OR = odds ratio; CI = confidence interval. (DOCX) [file pone.0253210.s004.docx]

| **S1 Table. Binary Logistic and Linear Regressions for Demographics** | | | | |
| --- | --- | --- | --- | --- |
| **Variable** | | | **Appropriateness ≥7***  **OR (95% CI)** | **Prioritization**  **β (95% CI)** |
| **Physician** | | |  | – |
| 1 | (N=88) | |  |  |
| 2 | (N=94) | | 1.70 (0.84 – 3.44) |  |
| 3 | (N=39) | | 14.25 (1.85 – 109.62) |  |
| 4 | (N=19) | | 3.19 (0.68 – 14.85) |  |
| 5 | (N=150) | | 4.74 (2.19 – 10.26) |  |
| 6 | (N=12) | | (0 – Infinity) |  |
| 7 | (N=65) | | 0.47 (0.24 – 0.92) |  |
| **Age** | |  | 0.98 (0.95 – 1.01) | -0.01 (-0.02 – 0.00) |
| **Age Decade** | |  |  |  |
| 40-49 | | (N=6) | (0 – Infinity) | 0.35 (-0.50 – 1.20) |
| 50-59 | | (N=58) | 1.77 (0.55 – 5.70) | 0.03 (-0.36 – 0.41) |
| 60-69 | | (N=153) | 0.86 (0.34 – 2.18) | -0.19 (-0.52 – 0.14) |
| 70-79 | | (N=205) | 0.80 (0.32 – 1.98) | -0.20 (-0.52 – 0.13) |
| 80-89 | | (N=45) |  |  |
| **Gender** | |  |  |  |
| Female | | (N=256) |  |  |
| Male | | (N=211) | 1.17 (0.70 – 1.95) | 0.04 (-0.14 – 0.22) |
| **Ethnicity** | |  |  |  |
| Europe | | (N=187) |  |  |
| Africa | | (N=17) | 2.77 (0.33 – 22.99) | -0.05 (-0.54 – 0.45) |
| Americas | | (N=130) | 0.79 (0.41 – 1.50) | 0.08 (-0.14 – 0.31) |
| Asia | | (N=130) | 0.94 (0.51 – 1.73) | -0.11 (-0.34 – 0.11) |
| **Annual Household Income** | |  |  |  |
| < $30 000 | | (N=79) |  |  |
| $30 000 - $49 999 | | (N=78) | 0.50 (0.21 – 1.18) | -0.43 (-0.74 – -0.13) |
| $50 000 - $69 999 | | (N=49) | 0.97 (0.34 – 2.80) | 0.00 (-0.35 – 0.35) |
| $70 000 + | | (N=103) | 0.72 (0.31 – 1.67) | -0.14 (-0.43 – 0.14) |
| **Education** | |  |  |  |
| ≤High School/Apprenticeship | | (N=221) |  |  |
| College/University | | (N=242) | 0.83 (0.50 – 1.39) | -0.05 (-0.23 – 0.13) |
| *Physician rating has been accounted for in each regression analysis for appropriateness  Statistically significant parameters are highlighted in yellow  OR = odds ratio; CI = confidence interval | | | | |
