# Supplementary material for: Physician and patient concordance in reporting of appropriateness and prioritization for cataract surgery
Source: PLoS One. 2021 Jun 25;16(6):e0253210. doi: 10.1371/journal.pone.0253210 (PMC8232411; doi:10.1371/journal.pone.0253210)
Supplement: S2 Table — *Physician rating has been accounted for in each regression analysis for appropriateness. Statistically significant parameters are highlighted in yellow (by overall model F-test and parameter specific t-test). eCAPS = electronic cataract appropriateness and prioritization system; BCVA = best-corrected visual acuity; OR = odds ratio; CI = confidence interval. (DOCX) [file pone.0253210.s005.docx]

| **S2 Table. Logistic and Linear Regressions for Clinical Criteria** | | | | | |
| --- | --- | --- | --- | --- | --- |
| **Variable** | | **Appropriateness ≥7***  **OR (95% CI)** | **Multiple Logistic Regression:**  **Appropriateness ≥7***  **OR (95% CI)** | **Prioritization**  **β (95% CI)** | **Multiple Linear Regression:**  **Prioritization**  **β (95% CI)** |
| **Study Eye Preoperative BCVA ≤20/50** | |  |  |  |  |
| >20/50 | (N=184) |  |  |  |  |
| ≤20/50 | (N=283) | 3.15 (1.82 – 5.48) | – | 0.70 (0.52 – 0.87) |  |
| **Fellow Eye Preoperative BCVA ≤20/50** | |  |  |  |  |
| >20/50 | (N=315) |  |  |  |  |
| ≤20/50 | (N=152) | 2.54 (1.38 – 4.68) | – | 0.29 (0.10 – 0.48) |  |
| **eCAPS Clinical Criteria** | | | | | |
| **Study Eye Preoperative BCVA** | |  |  |  |  |
| ≥20/30 | (N=86) |  |  |  |  |
| 20/40 – 20/50 | (N=170) | 2.40 (1.21 – 4.75) | 2.62 (1.21 – 5.68) | 0.27 (0.03 – 0.51) | 0.29 (0.47 – 0.54) |
| 20/60 – 20/150 | (N=119) | 5.34 (2.41 – 11.84) | 6.01 (2.34 – 15.45) | 0.89 (0.63 – 1.14) | 0.90 (0.63 – 1.17) |
| ≤20/200 | (N=91) | 7.74 (3.08 – 19.44) | 12.37 (4.00 – 38.23) | 0.96 (0.68 – 1.23) | 0.88 (0.59 – 1.17) |
| **Fellow Eye Preoperative BCVA** | |  |  |  |  |
| ≥20/30 | (N=207) |  |  |  |  |
| 20/40 – 20/50 | (N=172) | 1.83 (0.99 – 3.37) | 1.37 (0.66 – 2.83) | 0.03 (-0.17 – 0.23) | -0.07 (-0.26 – 0.13) |
| 20/60 – 20/150 | (N=62) | 2.06 (0.92 – 4.65) | 0.94 (0.35 – 2.48) | 0.26 (-0.02 – 0.54) | -0.11 (-0.38 – 0.17) |
| ≤20/200 | (N=23) | 6.14 (1.26 – 29.85) | 3.73 (0.65 – 21.34) | 0.75 (0.33 – 1.18) | 0.35 (-0.07 – 0.77) |
| **C.1 Anticipated Postoperative BCVA** | |  |  |  |  |
| Limited | (N=11) |  |  |  |  |
| Questionable | (N=40) | 4.28 (0.71 – 25.73) | 9.32 (1.15 – 75.76) | 0.44 (-0.22 – 1.10) | 0.50 (-0.14 – 1.15) |
| Good | (N=415) | 3.11 (0.69 – 14.05) | 13.22 (1.72 – 101.55) | 0.00 (-0.59 – 0.59) | 0.43 (-0.19 – 1.05) |
| **C.2 Anisometropia** | |  |  |  |  |
| No | (N=457) |  |  |  |  |
| Yes | (N=10) | 0.39 (0.07 – 2.33) | 0.33 (0.04 – 2.90) | 0.11 (-0.52 – 0.73) | -0.15 (-0.80 – 0.49) |
| **C.3 Monocular** | |  |  |  |  |
| No | (N=464) |  |  |  |  |
| Yes | (N=3) | (0 – Infinity) | (0 – Infinity) | 1.38 (0.26 – 2.51) | 0.72 (-0.36 – 1.79) |
| **C.4 Case Complexity** | |  |  |  |  |
| Routine | (N=422) |  |  |  |  |
| Complex | (N=41) | 0.76 (0.28 – 2.12) | 0.90 (0.26 – 3.07) | 0.33 (0.01 – 0.65) | 0.16 (-0.16 – 0.49) |
| **C.5 Presence of Ocular Comorbidity** | |  |  |  |  |
| No | (N=293) |  |  |  |  |
| Yes | (N=172) | 1.10 (0.60 – 2.01) | 0.95 (0.44 – 2.02) | 0.21 (0.02 – 0.39) | 0.12 (-0.07 – 0.32) |
| **C.6 Comorbidity Impact on Postoperative Improvement** | |  | – |  | – |
| None/Mild | (N=99) |  |  |  |  |
| Moderate | (N=55) | 1.94 (0.59 – 6.38) |  | 0.38 (0.04 – 0.71) |  |
| Severe | (N=15) | 0.70 (0.16 – 3.13) |  | -0.20 (-0.75 – 0.35) |  |
| **C.7 Comorbidity Impact on Timing of Surgery** | |  | – |  | – |
| None/Mild | (N=112) |  |  |  |  |
| Moderate | (N=49) | 1.09 (0.34 – 3.56) |  | 0.58 (0.25 – 0.92) |  |
| Severe | (N=8) | 0.62 (0.10 – 3.74) |  | 0.04 (-0.68 – 0.75) |  |
| **C.8 Cataract Impact on Comorbidity Treatment** | |  | – |  | – |
| None/Mild | (N=117) |  |  |  |  |
| Moderate | (N=43) | 3.09 (0.78 – 12.33) |  | 0.35 (0.01 – 0.70) |  |
| Severe | (N=9) | 1.58 (0.17 – 15.02) |  | 1.15 (0.48 – 1.82) |  |
| *Physician rating has been accounted for in each regression analysis for appropriateness  Statistically significant parameters are highlighted in yellow  eCAPS = electronic cataract appropriateness and prioritization system; BCVA = best-corrected visual acuity; OR = odds ratio; CI = confidence interval | | | | | |
