# Supplementary material for: Physician and patient concordance in reporting of appropriateness and prioritization for cataract surgery
Source: PLoS One. 2021 Jun 25;16(6):e0253210. doi: 10.1371/journal.pone.0253210 (PMC8232411; doi:10.1371/journal.pone.0253210)
Supplement: S3 Table — *Physician rating has been accounted for in each regression analysis for appropriateness. Statistically significant parameters are highlighted in yellow (by overall model F-test and parameter specific t-test). eCAPS = electronic cataract appropriateness and prioritization system; OR = odds ratio; CI = confidence interval; QoL = quality of life; VAS = visual analogue scale. (DOCX) [file pone.0253210.s006.docx]

| **S3 Table. Logistic and Linear Regressions for PROMs** | | | | | | |
| --- | --- | --- | --- | --- | --- | --- |
| **Variable** | | | **Appropriateness ≥7***  **OR (95% CI)** | **Multiple Logistic Regression:**  **Appropriateness ≥7***  **OR (95% CI)** | **Prioritization**  **β (95% CI)** | **Multiple Linear Regression:**  **Prioritization**  **β (95% CI)** |
| **eCAPS QoL** | | | | | | |
| **Q.1 Glare** | | |  |  |  |  |
| No | (N=159) | |  |  |  |  |
| Yes | (N=307) | | 1.55 (0.92 – 2.61) | 1.10 (0.58 – 2.08) | 0.03 (-0.16 – 0.22) | -0.07 (-0.27 – 0.12) |
| **Q.2 Night Driving Difficulty** | | |  |  |  |  |
| No | (N=218) | |  |  |  |  |
| Yes | (N=248) | | 2.02 (1.20 – 3.40) | 2.64 (1.38 – 5.05) | 0.16 (-0.01 – 0.34) | 0.26 (0.08 – 0.45) |
| **Q.3 Extent of Impairment in Visual Function** | | |  |  |  |  |
| None | | (N=126) |  |  |  |  |
| Mild/Moderate/Severe | | (N=340) | 1.49 (0.86 – 2.58) | 0.89 (0.46 – 1.73) | 0.07 (-0.13 – 0.28) | -0.16 (-0.35 – 0.04) |
| **Q.3 Extent of Impairment in Visual Function** | | |  | – |  | – |
| None | | (N=126) |  |  |  |  |
| Mild/Moderate | | (N=258) | 1.39 (0.78 – 2.47) |  | -0.04 (-0.25 – 0.16) |  |
| Severe | | (N=82) | 1.89 (0.82 – 4.34) |  | 0.44 (0.17 – 0.71) |  |
| **Q.4 Other Substantial Disabilities** | | |  |  |  |  |
| None | | (N=388) |  |  |  |  |
| Mild/Moderate/Severe | | (N=77) | 0.77 (0.40 – 1.47) | 0.69 (0.32 – 1.49) | -0.04 (-0.28 – 0.21) | -0.19 (-0.43 – 0.05) |
| **Q.4 Other Substantial Disabilities** | | |  | – |  | – |
| None | | (N=388) |  |  |  |  |
| Mild/Moderate | | (N=69) | 0.91 (0.45 – 1.84) |  | -0.06 (-0.31 – 0.20) |  |
| Severe | | (N=8) | 0.29 (0.06 – 1.37) |  | 0.12 (-0.58 – 0.82) |  |
| **Q.5 Safety and Injury Concerns** | | |  |  |  |  |
| None | | (N=340) |  |  |  |  |
| Mild/Moderate/Severe | | (N=126) | 1.61 (0.87 – 2.99) | 1.75 (0.78 – 3.94) | 0.25 (0.05 – 0.45) | -0.02 (-0.24 – 0.21) |
| **Q.5 Safety and Injury Concerns** | | |  | – |  | – |
| None | | (N=340) |  |  |  |  |
| Mild/Moderate | | (N=99) | 1.75 (0.88 – 3.51) |  | 0.19 (-0.03 – 0.41) |  |
| Severe | | (N=27) | 1.19 (0.38 – 3.77) |  | 0.47 (0.08 – 0.86) |  |
| **Q.6 Ability to Work/Care for Dependents** | | |  |  |  |  |
| None | | (N=377) |  |  |  |  |
| Mild/Moderate/Severe | | (84) | 1.44 (0.70 – 2.97) | 0.91 (0.35 – 2.39) | 0.44 (0.20 – 0.67) | 0.19 (-0.07 – 0.45) |
| **Q.6 Ability to Work/Care for Dependents** | | |  | – |  | – |
| None | | (N=377) |  |  |  |  |
| Mild/Moderate | | (N=63) | 1.36 (0.62 – 3.00) |  | 0.27 (0.01 – 0.53) |  |
| Severe | | (N=21) | 1.82 (0.39 – 8.54) |  | 0.93 (0.50 – 1.35) |  |
| **Q.7 Ability to Take Care of Local Errands** | | |  |  |  |  |
| None | | (N=369) |  |  |  |  |
| Mild/Moderate/Severe | | (N=97) | 2.29 (1.09 – 4.83) | 2.44 (0.93 – 6.41) | 0.54 (0.32 – 0.75) | 0.37 (0.13 – 0.62) |
| **Q.7 Ability to Take Care of Local Errands** | | |  | – |  | – |
| None | | (N=369) |  |  |  |  |
| Mild/Moderate | | (N=80) | 2.43 (1.07 – 5.52) |  | 0.44 (0.21 – 0.68) |  |
| Severe | | (N=17) | 1.74 (0.36 – 8.46) |  | 0.98 (0.50 – 1.45) |  |
| **Q.8 Ability to Take Care of Household Business** | | |  |  |  |  |
| None | | (N=414) |  |  |  |  |
| Mild/Moderate/Severe | | (N=52) | 1.39 (0.58 – 3.35) | 1.04 (0.28 – 3.91) | 0.36 (0.08 – 0.65) | -0.04 (-0.37 – 0.30) |
| **Q.8 Ability to Take Care of Household Business** | | |  | – |  | – |
| None | | (N=414) |  |  |  |  |
| Mild/Moderate | | (N=44) | 1.23 (0.50 – 3.00) |  | 0.22 (-0.08 – 0.53) |  |
| Severe | | (N=8) | (0 – Infinity) |  | 1.13 (0.44 – 1.82) |  |
| **Q.9 Taking Care of Your Own Health** | | |  |  |  |  |
| None | | (N=427) |  |  |  |  |
| Mild/Moderate/Severe | | (N=39) | 1.21 (0.49 – 2.99) | 0.63 (0.19 – 2.12) | 0.41 (0.08 – 0.74) | 0.06 (-0.30 – 0.42) |
| **Q.9 Taking Care of Your Own Health** | | |  | – |  | – |
| None | | (N=427) |  |  |  |  |
| Mild/Moderate | | (N=34) | 1.11 (0.45 – 2.77) |  | 0.28 (-0.07 – 0.62) |  |
| Severe | | (N=5) | (0 – Infinity) |  | 1.32 (0.45 – 2.19) |  |
| **Q.10 Ability to Provide Assistance to Others** | | |  |  |  |  |
| None | | (N=403) |  |  |  |  |
| Mild/Moderate/Severe | | (N=63) | 1.36 (0.63 – 2.92) | 1.95 (0.55 – 6.90) | 0.38 (0.12 – 0.64) | 0.05 (-0.29 – 0.38) |
| **Q.10 Ability to Provide Assistance to Others** | | |  | – |  | – |
| None | | (N=403) |  |  |  |  |
| Mild/Moderate | | (N=52) | 1.26 (0.56 – 2.81) |  | 0.28 (-0.01 – 0.56) |  |
| Severe | | (N=11) | 2.33 (0.26 – 20.82) |  | 0.85 (0.26 – 1.44) |  |
| **Q.11 Ability to Participate in Social Life** | | |  |  |  |  |
| None | | (N=390) |  |  |  |  |
| Mild/Moderate/Severe | | (N=75) | 1.40 (0.66 – 2.97) | 0.51 (0.18 – 1.50) | 0.45 (0.21 – 0.70) | 0.17 (-0.13 – 0.46) |
| **Q.11 Ability to Participate in Social Life** | | |  | – |  | – |
| None | | (N=390) |  |  |  |  |
| Mild/Moderate | | (N=62) | 1.46 (0.64 – 3.32) |  | 0.28 (0.02 – 0.55) |  |
| Severe | | (N=13) | 1.19 (0.24 – 5.99) |  | 1.25 (0.71 – 1.79) |  |
| **Q.12 Take Part in Active Recreational Activities** | | |  |  |  |  |
| None | | (N=353) |  |  |  |  |
| Mild/Moderate/Severe | | (N=111) | 0.86 (0.47 – 1.56) | 0.72 (0.31 – 1.66) | 0.17 (-0.05 – 0.38) | -0.08 (-0.32 – 0.16) |
| **Q.12 Take Part in Active Recreational Activities** | | |  | – |  | – |
| None | | (N=353) |  |  |  |  |
| Mild/Moderate | | (N=80) | 0.69 (0.36 – 1.33) |  | 0.04 (-0.20 – 0.28) |  |
| Severe | | (N=31) | 1.83 (0.51 – 6.58) |  | 0.49 (0.13 – 0.86) |  |
| **eCAPS QoL Aggregate Score** | |  |  |  |  |  |
| As continuous variable: | |  | 1.07 (0.97 – 1.18) | – | 0.08 (0.05 – 0.11) | – |
| **Catquest-9SF** | | | | | | |
| **A. Difficulty in Everyday Life Due to Vision** | | |  |  |  |  |
| No difficulty | | (N=82) |  |  |  |  |
| Difficulty | | (N=220) | 1.17 (0.59 – 2.32) | 0.65 (0.26 – 1.65) | 0.05 (-0.20 – 0.29) | -0.13 (-0.44 – 0.18) |
| **A. Difficulty in Everyday Life Due to Vision** | | |  | – |  | – |
| No difficulty | | (N=82) |  |  |  |  |
| Some difficulty | | (N=169) | 0.88 (0.43 – 1.79) |  | -0.06 (-0.32 – 0.19) |  |
| Great difficulty | | (N=41) | 2.95 (0.88 – 9.86) |  | 0.33 (-0.03 – 0.69) |  |
| Very great difficulty | | (N=10) | 3.37 (0.38 – 30.17) |  | 0.75 (0.12 – 1.38) |  |
| Cannot decide | | (N=0) | - |  | - |  |
| **B. Satisfied with Present Vision** | | |  |  |  |  |
| Satisfied | | (N=92) |  |  |  |  |
| Dissatisfied | | (N=210) | 1.18 (0.59 – 2.34) | 0.90 (0.34 – 2.39) | 0.05 (-0.19 – 0.29) | -0.10 (-0.39 – 0.19) |
| **B. Satisfied with Present Vision** | | |  | – |  | – |
| Very satisfied | | (N=12) |  |  |  |  |
| Fairly satisfied | | (N=80) | 0.85 (0.18 – 4.15) |  | -0.28 (-0.86 – 0.31) |  |
| Fairly dissatisfied | | (N=147) | 0.79 (0.17 – 3.63) |  | -0.30 (-0.87 – 0.27) |  |
| Very dissatisfied | | (N=63) | 2.72 (0.47 – 15.81) |  | 0.07 (-0.52 – 0.67) |  |
| Cannot decide | | (N=0) | - |  | - |  |
| **C.1 Reading Text in Newspaper** | | |  |  |  |  |
| No difficulty | | (N=91) |  |  |  |  |
| Difficulty | | (N=216) | 0.97 (0.49 - 1.93) | 0.70 (0.24 – 1.99) | 0.12 (-0.12 – 0.35) | -0.03 (-0.34 – 0.28) |
| **C.1 Reading Text in Newspaper** | | |  | – |  | – |
| No difficulty | | (N=91) |  |  |  |  |
| Some difficulty | | (N=116) | 0.81 (0.38 – 1.72) |  | 0.07 (-0.19 – 0.33) |  |
| Great difficulty | | (N=59) | 1.28 (0.47 – 3.45) |  | 0.18 (-0.13 – 0.50) |  |
| Very great difficulty | | (N=41) | 1.20 (0.42 – 3.44) |  | 0.14 (-0.21 – 0.50) |  |
| Cannot decide | | (N=0) | - |  | - |  |
| **C.2 Recognizing Faces** | | |  |  |  |  |
| No difficulty | | (N=215) |  |  |  |  |
| Difficulty | | (N=93) | 1.81 (0.87 – 3.75) | 1.54 (0.61 – 3.85) | 0.35 (0.12 – 0.58) | 0.25 (-0.02 – 0.52) |
| **C.2 Recognizing Faces** | | |  | – |  | – |
| No difficulty | | (N=215) |  |  |  |  |
| Some difficulty | | (N=64) | 2.45 (1.04 – 5.79) |  | 0.38 (0.11 – 0.64) |  |
| Great difficulty | | (N=19) | 0.58 (0.14 – 2.44) |  | 0.05 (-0.39 – 0.50) |  |
| Very great difficulty | | (N=10) | 1.53 (0.15 – 15.61) |  | 0.73 (0.13 – 1.33) |  |
| Cannot decide | | (N=0) | - |  | - |  |
| **C.3 Seeing Prices** | | |  |  |  |  |
| No difficulty | | (N=105) |  |  |  |  |
| Difficulty | | (N=200) | 1.61 (0.84 – 3.08) | 2.15 (0.76 – 6.08) | 0.29 (0.07 – 0.52) | 0.39 (0.07 – 0.71) |
| **C.3 Seeing Prices** | | |  | – |  | – |
| No difficulty | | (N=105) |  |  |  |  |
| Some difficulty | | (N=118) | 1.35 (0.66 – 2.79) |  | 0.26 (0.00 – 0.51) |  |
| Great difficulty | | (N=45) | 1.65 (0.52 – 5.20) |  | 0.35 (0.02 – 0.69) |  |
| Very great difficulty | | (N=37) | 2.61 (0.86 – 7.91) |  | 0.34 (-0.02 – 0.70) |  |
| Cannot decide | | (N=0) | - |  | - |  |
| **C.4 Seeing to Walk on Uneven Ground** | | |  |  |  |  |
| No difficulty | | (N=174) |  |  |  |  |
| Difficulty | | (N=127) | 2.31 (1.17 – 4.55) | 1.77 (0.71 – 4.39) | 0.34 (0.12 – 0.55) | 0.28 (0.02 – 0.55) |
| **C.4 Seeing to Walk on Uneven Ground** | | |  | – |  | – |
| No difficulty | | (N=174) |  |  |  |  |
| Some difficulty | | (N=80) | 2.59 (1.15 – 5.87) |  | 0.26 (0.02 – 0.51) |  |
| Great difficulty | | (N=36) | 1.69 (0.60 – 4.74) |  | 0.32 (-0.02 – 0.65) |  |
| Very great difficulty | | (N=11) | 3.20 (0.37 – 27.79) |  | 0.95 (0.39 – 1.52) |  |
| Cannot decide | | (N=0) | - |  | - |  |
| **C.5 Seeing to do Handiwork** | | |  |  |  |  |
| No difficulty | | (N=168) |  |  |  |  |
| Difficulty | | (N=127) | 1.59 (0.78 – 3.25) | 1.44 (0.43 – 4.90) | 0.10 (-0.12 – 0.32) | -0.12 (-0.43 – 0.20) |
| **C.5 Seeing to do Handiwork** | | |  | – |  | – |
| No difficulty | | (N=168) |  |  |  |  |
| Some difficulty | | (N=83) | 1.60 (0.71 – 3.61) |  | -0.02 (-0.27 – 0.24) |  |
| Great difficulty | | (N=28) | 0.81 (0.24 – 2.74) |  | 0.37 (-0.01 – 0.75) |  |
| Very great difficulty | | (N=16) | (0 – Infinity) |  | 0.21 (-0.28 – 0.71) |  |
| Cannot decide | | (N=0) | - |  | - |  |
| **C.6 Reading Subtitles on TV** | | |  |  |  |  |
| No difficulty | | (N=108) |  |  |  |  |
| Difficulty | | (N=196) | 1.79 (0.93 – 3.44) | 1.44 (0.54 – 3.89) | 0.10 (-0.13 – 0.32) | -0.18 (-0.48 – 0.12) |
| **C.6 Reading Subtitles on TV** | | |  | – |  | – |
| No difficulty | | (N=108) |  |  |  |  |
| Some difficulty | | (N=115) | 2.00 (0.95 – 4.23) |  | 0.08 (-0.18 – 0.33) |  |
| Great difficulty | | (N=57) | 1.42 (0.56 – 3.63) |  | -0.01 (-0.32 – 0.30) |  |
| Very great difficulty | | (N=24) | 1.78 (0.51 – 6.24) |  | 0.46 (0.03 – 0.88) |  |
| Cannot decide | | (N=0) | - |  | - |  |
| **C.7 Seeing to Engage in an Activity/Hobby** | | |  |  |  |  |
| No difficulty | | (N=173) |  |  |  |  |
| Difficulty | | (N=132) | 1.29 (0.66 – 2.51) | 0.60 (0.16 – 2.16) | 0.22 (0.00 – 0.43) | 0.13 (-0.20 – 0.46) |
| **C.7 Seeing to Engage in an Activity/Hobby** | | |  | – |  | – |
| No difficulty | | (N=173) |  |  |  |  |
| Some difficulty | | (N=88) | 0.83 (0.39 – 1.77) |  | 0.13 (-0.12 – 0.37) |  |
| Great difficulty | | (N=29) | 3.71 (0.79 – 17.46) |  | 0.30 (-0.08 – 0.68) |  |
| Very great difficulty | | (N=15) | 2.53 (0.51 – 12.43) |  | 0.59 (0.09 – 1.09) |  |
| Cannot decide | | (N=0) | - |  | - |  |
| **Catquest-9SF Preoperative Score** | | | 1.23 (0.99 – 1.53) | – | 0.10 (0.03 – 0.17) | – |
| **EQ-5D** | | | | | | |
| **Q.1 Mobility** | |  |  |  |  |  |
| No problem | | (N=201) |  |  |  |  |
| Problem | | (N=107) | 1.08 (0.55 – 2.13) | – | 0.11 (-0.12 – 0.33) | – |
| **Q.1 Mobility** | | |  | – |  | – |
| No problem | | (N=201) |  |  |  |  |
| Slight problem | | (N=49) | 1.32 (0.50 – 3.49) |  | 0.03 (-0.27 – 0.32) |  |
| Moderate problem | | (N=45) | 0.98 (0.40 – 2.42) |  | -0.00 (-0.31 – 0.31) |  |
| Severe problem | | (N=10) | 0.67 (0.10 – 4.52) |  | 0.67 (0.07 – 1.28) |  |
| Unable | | (N=3) | 1.29 (0.11 – 15.43) |  | 1.21 (0.12 – 2.29) |  |
| **Q.2 Self-Care** | |  |  |  |  |  |
| No problem | | (N=285) |  |  |  |  |
| Problem | | (N=22) | 0.97 (0.29 – 3.20) | – | 0.28 (-0.14 – 0.69) | – |
| **Q.2 Self-Care** | | |  | – |  | – |
| No problem | | (N=285) |  |  |  |  |
| Slight problem | | (N=6) | 0.21 (0.02 – 2.17) |  | -0.30 (-1.07 – 0.47) |  |
| Moderate Problem | | (N=9) | 1.69 (0.16 – 17.32) |  | 0.41 (-0.22 – 1.05) |  |
| Severe problem | | (N=3) | 0.78 (0.05 – 13.11) |  | 1.19 (0.10 – 2.28) |  |
| Unable | | (N=4) | (0 – Infinity) |  | 0.15 (-0.79 – 1.10) |  |
| **Q.3 Usual Activities** | |  |  |  |  |  |
| No problem | | (N=206) |  |  |  |  |
| Problem | | (N=103) | 1.26 (0.63 – 2.54) | – | 0.20 (-0.02 – 0.43) | – |
| **Q.3 Usual Activities** | | |  | – |  | – |
| No problem | | (N=206) |  |  |  |  |
| Slight problem | | (N=52) | 0.81 (0.34 – 1.96) |  | -0.07 (-0.35 – 0.22) |  |
| Moderate problem | | (N=37) | 1.97 (0.60 – 6.43) |  | 0.50 (0.16 – 0.83) |  |
| Severe problem | | (N=11) | 1.69 (0.31 – 9.22) |  | 0.35 (-0.22 – 0.93) |  |
| Unable | | (N=3) | (0 – Infinity) |  | 0.74 (-0.34 – 1.82) |  |
| **Q.4 Pain/Discomfort** | |  |  |  |  |  |
| None | | (N=161) |  |  |  |  |
| Present | | (N=148) | 1.27 (0.67 – 2.39) | – | -0.01 (-0.23 – 0.20) | – |
| **Q.4 Pain/Discomfort** | | |  | – |  | – |
| None | | (N=161) |  |  |  |  |
| Slight | | (N=67) | 1.73 (0.74 – 4.02) |  | 0.05 (-0.23 – 0.32) |  |
| Moderate | | (N=61) | 1.12 (0.49 – 2.55) |  | 0.04 (-0.24 – 0.33) |  |
| Severe | | (N=17) | 0.56 (0.15 – 2.06) |  | -0.54 (-1.02 – -0.07) |  |
| Extreme | | (N=3) | (0 – Infinity) |  | 0.52 (-0.57 – 1.62) |  |
| **Q.5 Anxiety/Depression** | |  |  |  |  |  |
| None | | (N=202) |  |  |  |  |
| Present | | (N=107) | 0.69 (0.35 – 1.35) | – | -0.03 (-0.26 – 0.19) | – |
| **Q.5 Anxiety/Depression** | |  |  | – |  | – |
| None | | (N=202) |  |  |  |  |
| Slight | | (N=69) | 0.67 (0.31 – 1.46) |  | -0.10 (-0.36 – 0.17) |  |
| Moderate | | (N=33) | 0.69 (0.21 – 2.25) |  | 0.09 (-0.26 – 0.45) |  |
| Severe | | (N=4) | 0.89 (0.07 – 12.03) |  | -0.13 (-1.09 – 0.82) |  |
| Extreme | | (N=1) | (0 – Infinity) |  | 0.50 (-1.40 – 2.40) |  |
| **Reported Health Status (VAS)** | | |  | – |  | – |
| As continuous variable: | | | 1.00 (0.98 – 1.02) |  | -0.01 (-0.01 – 0.00) |  |
| **EQ-5D Preoperative Score** | | | 0.81 (0.09 – 7.18) | – | -0.38 (-1.15 – 0.40) | – |
| *Physician rating has been accounted for in each regression analysis for appropriateness  Statistically significant parameters are highlighted in yellow  eCAPS = electronic cataract appropriateness and prioritization system; OR = odds ratio; CI = confidence interval; QoL = quality of life; VAS = visual analogue scale | | | | | | |
